# Electromechanical Properties of Smart Vitrimers Reinforced with Carbon Nanotubes for SHM Applications

**DOI:** 10.3390/s24030806

**Published:** 2024-01-26

**Authors:** Javier Gómez-Sánchez, Xoan F. Sánchez-Romate, Francisco Javier Espadas, Silvia G. Prolongo, Alberto Jiménez-Suárez

**Affiliations:** 1 Materials Science and Engineering Area, Escuela Superior de Ciencias Experimentales y Tecnología, Universidad Rey Juan Carlos, Calle Tulipán s/n, Móstoles, 28933 Madrid, Spain; xoan.fernandez.sanchezromate@urjc.es (X.F.S.-R.); fj.espadas.2017@alumnos.urjc.es (F.J.E.); silvia.gonzalez@urjc.es (S.G.P.); 2 Instituto de Tecnologías para la Sostenibilidad, Universidad Rey Juan Carlos, Calle Tulipán s/n, Móstoles, 28933 Madrid, Spain

**Keywords:** vitrimer, disulfide bonds, epoxy, carbon nanotubes, strain sensing, electrical properties, mechanical properties

## Abstract

The Structural Health Monitoring (SHM) capabilities of a well-studied self-healing epoxy resin based on disulfide bonds, through the addition of carbon nanotubes (CNTs), are studied. Since these materials demonstrated, in recent works, a high dependency of the dynamic hardener content on the repair performance, this study aimed to analyze the effect of the vitrimeric chemistry on the electromechanical properties by studying different 2-aminophenyl disulfide (2-AFD) hardener and CNT contents. The electrical conductivity increases with both the CNT and AFD contents, in general. Moreover, an excess of AFD close to the stoichiometric ratio with a low CNT content improved the tensile strength by 45%, while higher AFD contents promoted its detriment by 41% due to a reduced crosslinking density. However, no significant difference in the mechanical properties was observed at a higher CNT content, regardless of the AFD ratio. The developed materials demonstrate a robust electromechanical response at quasi-static conditions. The sensitivity significantly increases at higher AFD ratios, from 0.69 to 2.22 for the 0.2 wt.%. CNT system, which is advantageous due to the enhanced repair performance of these vitrimeric materials with a higher hardener content. These results reveal the potential use of self-healing vitrimers as integrated SHM systems capable of detecting damages and self-repairing autonomously.

## 1. Introduction

The use of Fiber-Reinforced Polymer (FRP) composites with thermosetting matrices for high-performance applications is justified by the resin’s high crosslink density, high rigidity, good wettability, and excellent adhesion, among other properties [1]. However, their brittle behavior limits their applicability under certain loading and environmental conditions [2]. Moreover, unlike conventional alloys, composites are anisotropic or orthotropic materials in which damages are difficult to predict and, due to the highly crosslinked structure of thermosets, difficult to repair [3]. These factors promote a global reduction in the safety of the composite structure [4].

Nowadays, most repairing methods are based on the incorporation of patches after removing the damaged area [5]. Here, the main issue is the reliability of these patches, especially when using adhesives [6]. Therefore, this fact usually leads to the need to use rivets, with an associated weight increase [7].

For this reason, the incorporation of aromatic disulfide bonds into thermosets to provide them with self-healing functionality has attracted considerable attention due to their ability to repair damages and cracks, restoring the original properties of the material and, thereby, extending its lifetime [8]. The resulting polymers, so-called vitrimers, are based on dynamic covalent bonds that reversibly break and reform when subjected to an external stimulus, such as an activation temperature [9]. These exchange reactions between adjacent disulfide bonds trigger a decrease in the viscosity, permitting the polymer to gain a high molecular mobility state and flow [10].

Although the repair phenomena of these systems have been demonstrated in previous investigations [11], they cannot be categorized as smart materials since, by definition [12], they should react and respond to structural changes (e.g., damages) autonomously [13] and be assessed correctly and in time without reducing the safety and reliability of the structure to be addressed. Therefore, the development of Non-Destructive Testing (NDT) techniques is necessary to enhance the quality of the structures prior to and after their manufacturing [14]. Nevertheless, conventional NDT techniques do not offer continuous information on the health of a structure because they are performed in a particular period and not continuously [15].

Hence, to improve the safety of composite materials, it is necessary to implement a continuous monitoring method to detect damages: the so-called Structural Health Monitoring (SHM) system [16]. The SHM technology aims to assess the performance of a structure by detecting, identifying, and quantifying damage at an early stage through integrated sensor networks [17].

Traditional SHM techniques include metallic strain gauges [18], metal oxide films [19], piezoelectric sensors [20], and fiber optic sensors [21]. However, they are not sensitive often enough to properly monitor low strain levels, and the size and weight of the required systems make them difficult to use in an operational structure [22]. In this regard, carbon nanotubes (CNTs) have been investigated in recent years for SHM applications. Carbon-based nanocomposites provide several advantages by being directly embedded in the material, making them more sensitive to damage and strain field than an ex situ sensor would be, thus showing a much higher gauge factor (GF) [23,24].

The electrical properties of the CNTs are affected by three main factors: the intrinsic resistivity of carbon nanoparticles, which changes when subjected to mechanical strain; the contact resistance between adjacent nanoparticles, where the appearance and extent of the damage disrupt the electrical pathways and induce a variation in the electrical resistance; and the tunneling effect between neighboring nanoparticles, where the increase in distance between them with applied strain results in an exponential increase in tunneling resistance [25,26,27]. Therefore, the whole material itself acts as a sensor and detects both the strain and failure in real-time [28].

In this regard, this study aims to develop a self-healing epoxy resin doped with carbon nanotubes with SHM capabilities. The sensing properties, together with the ability to self-heal from damages, will enhance safety, reduce the intervals of maintenance in such materials, and increase the lifetime of the final product. To achieve this, the resin will be composed of a disulfide bond dynamic hardener, since they are increasingly being used for research purposes due to their high network rearrangement when exposed to a thermal stimulus. Even though 4-aminophenil disulfide (4-AFD) is gaining attention in recent literature reviews, 2-AFD was selected for this work, which is an isomer of the 4-AFD that is rarely used in research works. The reason behind this selection was its lower price, which is half of that of 4-AFD [29], making it an attractive solution for industry escalation. Moreover, the resin mixtures were prepared with different hardener proportions: stoichiometric, and with AFD excesses of 10% and 20% over the stoichiometric proportion. The use of higher concentrations of this isomer derives from previous research employing similar formulations that have incorporated an excess of the hardener to achieve the self-healing properties of interest [30]. Indeed, Luzuriaga et al. [31] demonstrated that a higher presence of free amines decreases the relaxation time, the glass transition temperature (T_g_), and the activation energy (E_a_) of the disulfide exchange reactions, indicating a higher network mobility effect of the non-stoichiometric systems. Furthermore, Benazzo et al. [32] performed a comparison of the self-healing performance of a DGEBA/AFD mixture in both stoichiometric and non-stoichiometric (20% of AFD excess) conditions via mechanical tests. The results showed a higher self-healing efficiency in the non-stoichiometric mixture (88.6%) compared to the stoichiometric system (50.2%). Moreover, two different concentrations of CNT (0.1 wt.% and 0.2 wt.%) were explored since the percolation threshold for CNT-doped epoxy resins has been demonstrated to be close to 0.1 wt.% CNTs. No further CNT contents were investigated since the sensitivity would be decreased [33]. In this way, the developed smart vitrimers were analyzed under quasi-static conditions to ensure an accurate measurement of the effect of both amine and CNT content in terms of electrical, via DC conductivity measurements, and electromechanical properties. In this context, tensile and three-point bending tests were performed with simultaneous electrical resistance recording to assess the strain monitoring capabilities.

## 2. Materials and Methods

### 2.1. Materials

The epoxy resin was composed of bisphenol A diglycidyl ether (DGEBA) as the monomer and 2-aminophenyl disulfide (AFD) as the dynamic hardener. Chemicals were supplied by Merck Life Sciences (Barcelona, Spain) and Tokyo Chemical Industry (TCI, Tokyo, Japan), respectively.

The multi-walled carbon nanotubes (MWCNTs) used in this work were NC7000, supplied by Nanocyl (Sambreville, Belgium), with an average length of 1.5 μm and an outer diameter of 9.5 nm.

### 2.2. Synthesis

The CNTs were first manually mixed with the DGEBA monomer and then dispersed through a three-roll milling process using an EXAKT 80E mini calender, from EXAKT Technologies Inc. (Oklahoma City, OK, USA). The process involved 7 cycles with a gradual reduction in the distance between adjacent rolls. These rolls rotated in opposite directions at a constant speed. The parameters used are shown in Table 1 and are derived from previous studies [34]. Once the dispersion was completed, the mixture was degassed at 80 °C for 15 min. The 2-AFD hardener was added in different proportions: stoichiometric, R = 1.0; and with an AFD excess, R = 1.1, R = 1.2, that is, 10%, and 20% AFD excess over the stochiometric proportion, respectively. The mixture was magnetically stirred for 5 min, maintaining a temperature of 80 °C, until phase miscibility occurred. Finally, it was poured into a metallic mold and cured following the optimized curing cycle of a previous research study [35], i.e., at 160 °C for 6 h in a convective oven.

### 2.3. Electrical Characterization

The electrical properties were characterized following ASTM D257 [36], using a Source Measurement Unit (SMU) from Keithley Instrument Inc. mod. 2410 (Cleveland, OH, USA). Four specimens of 10 × 10 × 1 mm^3^ were tested for each condition. The electrical resistance, R, was determined as the slope of the I–V curves, which showed an Ohmic behavior, with an applied voltage ranging from 0 to 20 and 0 to 100 V for samples with high and low conductivity, respectively. The electrical resistivity was determined considering the sample geometry by applying Equation (1):

(1) $$\rho =\frac{R\cdot A}{L},$$

where ρ is the electrical resistivity, *L* is the distance between the electrodes, and *A* is their contact area.

### 2.4. Strain Sensing Test via Electrical Measurements

Strain monitoring tests were conducted by performing tensile and three-point bending tests while simultaneously monitoring the electrical response of the specimens. These tests were carried out using a Zwick Z100 (Zwick-Roell, Ulm, Germany) universal tensile machine following standards ASTM D638 [37] and ASTM D790 [38], respectively. Tensile specimens were tested at a rate of 5 mm/min, while bending tests were first conducted at 1 mm/min up to 0.7% strain to determine the flexural modulus, and then at 10.05 mm/min up to failure to obtain the flexural strength.

**Figure 1:**
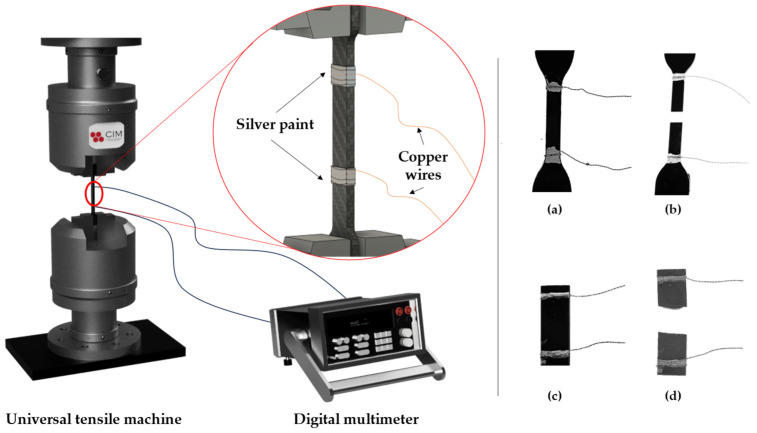
Scheme of the tensile electromechanical test, and real images for the tensile (**a**,**b**) and bending (**c**,**d**) specimens prior to and after the test, respectively.

The electrical monitoring was recorded simultaneously with electrical resistance measurements between two copper electrodes, which were attached to the test specimens with silver ink, using an Agilent 34410 A module (Agilent Technologies, Santa Clara, CA, USA), as shown in the schematics of Figure 1.

The strain sensitivity or gauge factor (GF) of the specimens was determined following Equation (2):

(2) $$GF=\frac{∆R/R_{0}}{\varepsilon }$$

where ∆*R/R*_0_ is the change in the normalized resistance, and *ε* is the applied strain.

### 2.5. Microstructural Characterization

Microstructural studies of the fractured surfaces under tensile conditions were performed via Scanning Electron Microscopy (SEM), using a S3400N machine from Hitachi Global (Tokyo, Japan).

Moreover, an analysis of the fracture surface under cryogenic conditions was carried out to characterize the CNT dispersion using a Field Emission Gun SEM (FEG-SEM) Nova NanoSEM 230 apparatus from Philips (Amsterdam, The Netherlands).

The samples were previously coated with a sputtering of gold to ensure a proper observation.

## 3. Results

In this section, first, an analysis of electrical properties under DC conductivity is carried out to shed light on how CNT and AFD ratios affect the electrical network. Then, the electromechanical properties of the developed smart materials are assessed under tensile and bending loads.

### 3.1. Electrical Conductivity

**Figure 2:**
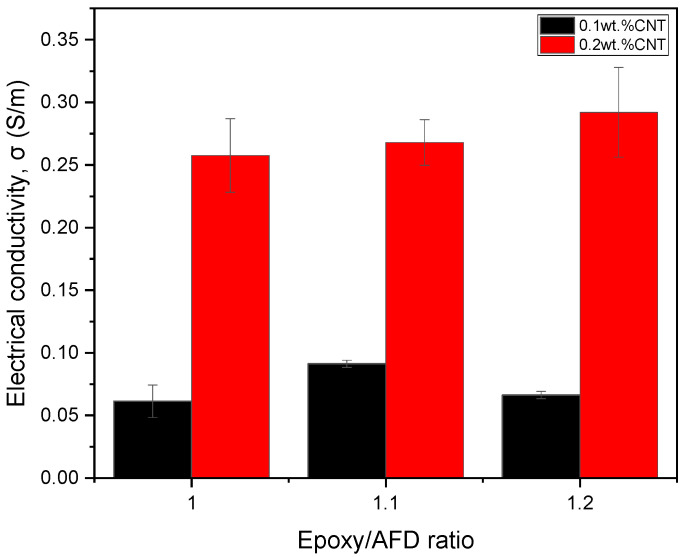
Electrical conductivity plots as a function of the epoxy/AFD ratio and CNT content.

Figure 2 shows the electrical conductivity values for the different CNT-AFD conditions. Here, on the one side, it can be observed that the electrical conductivity increases with CNT content, as expected, due to a higher number of electrical pathways. Moreover, the enhancement is quite significant, which is indicative of a partially good CNT dispersion as the saturation of the electrical network would not be achieved [39].

**Figure 3:**
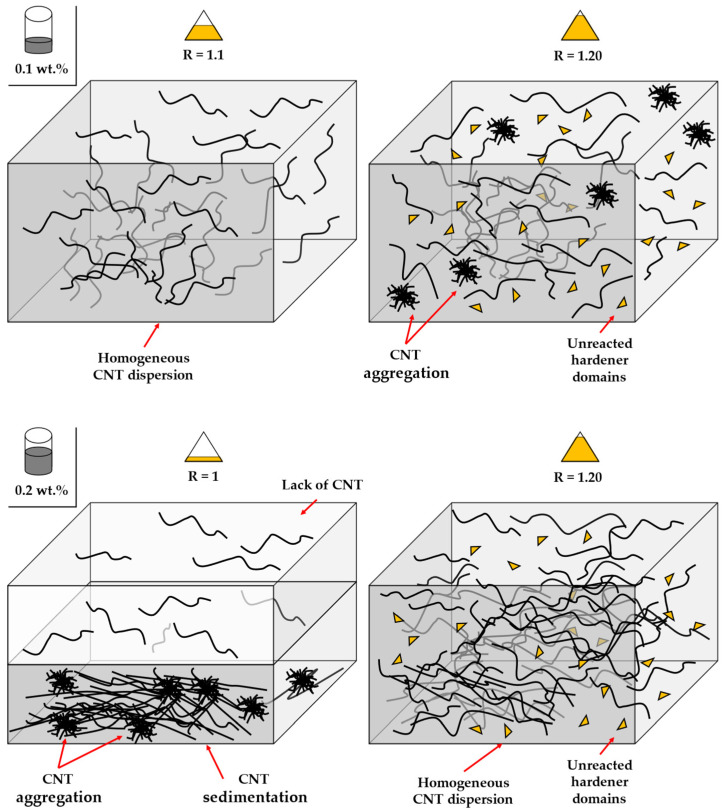
Schematics of the CNT and AFD influence on CNT distribution.

On the other side, the behavior of the electrical conductivity with AFD content is significantly more complex. In this regard, two effects may be taking part, shown in the schematics of Figure 3: the presence of unreacted domains that act as insulating areas with a lack of CNTs, which would promote a higher aggregation of the CNTs in the other regions; and the higher viscosity of the AFD, which would induce lower sedimentation during curing. The first effect would act by decreasing the electrical conductivity, whereas the second one would act in the opposite direction.

**Figure 4:**
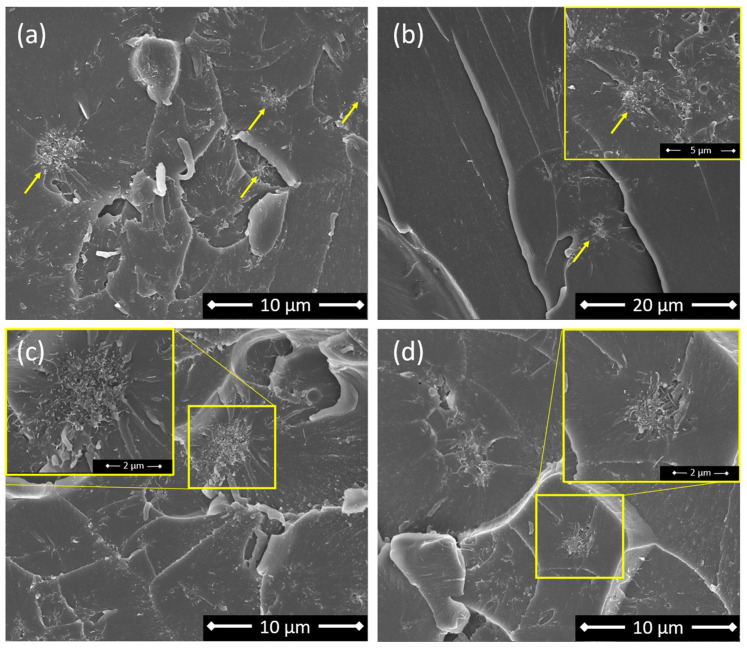
FEG-SEM micrographs of the cross-section of specimens with a (**a**) stoichiometric content of AFD (R = 1) and 0.1 wt.% CNT (**b**) 10% of AFD excess (R = 1.1) and 0.1 wt.% CNT; (**c**) stoichiometric content of AFD (R = 1) and 0.2 wt.% CNT; and (**d**) 20% of AFD excess (R = 1.2) and 0.2 wt.% CNT; marked in yellow and zoomed in on a region with agglomerated CNTs.

In the first case, linked to a low CNT content (i.e., 0.1 wt.% CNT), the addition of 10% of AFD excess in the polymer causes an increase in the electrical conductivity when compared to the stoichiometric mixture. However, a drop in this value occurs when this excess is increased to 20%. This behavior may be explained by the increased epoxy conversion achieved due to a higher proportion of amines [40]. When the mixture contains an amine/epoxy ratio slightly higher than R = 1, as in the case of the 10% of AFD excess, the reaction between amines and epoxy groups is statistically facilitated and all the amines of the hardener react, giving fully reacted hardener units. In the 20% AFD excess, all the primary amine hydrogens disappear, giving a higher epoxy conversion, but epoxide-secondary amine hydrogen addition occurs. As a result, the crosslinked material is essentially composed of chain segments with partially reacted hardener units. These unreacted domains occupy and reduce the free volume available for CNTs in the matrix, creating segregated CNT pathways and leading to their aggregation in other regions [41]. As a result, the dispersion efficiency is reduced, and the regions with partially reacted hardener units act as insulating areas, which reduce the overall electrical conductivity. This effect can be observed in Figure 4a,b, where the general view of the stoichiometric content shows a higher number of CNT agglomerates, whereas the 10% of AFD excess demonstrates a better dispersion with the presence of single small CNT agglomerates, leading to higher electrical conductivity.

In the second case (0.2 wt.% CNT), since CNTs tend to aggregate easily because of their low level of bending stiffness and van der Waals forces [42], the sedimentation effect of the CNT suspension becomes more prevalent due to the presence of more aggregated regions. However, increasing the AFD content increases the viscosity of the mixture, as it is a powdered solute, thus reducing this sedimentation effect. This rationale explains the larger CNT agglomerate size, shown in Figure 4c, for the stoichiometric mixture compared to the one shown in in Figure 4d for a 20% AFD excess, resulting in higher electrical conductivity in the latter, as it presents a much more efficient electrical network.

### 3.2. Mechanical and Strain-Sensing Analysis

#### 3.2.1. Tensile Tests

Once the electrical properties and their correlation with the CNT distribution were analyzed, the influence of the AFD and CNT content on the mechanical and strain-sensing properties of the vitrimers was examined via tensile and flexural tests, as previously described. Table 2 shows the values of some of the main mechanical properties in tensile conditions. Here, at lower CNT contents (0.1 wt.% CNT), the addition of 10% of AFD excess causes an increase in tensile strength and Young’s modulus when compared to the stoichiometric mixture, while a drop in these values occurs when this excess is increased to 20%. This effect is related to the previous rationale of the AFD influence on the electrical behavior. The 10% of AFD excess system results in fully reacted hardener units, while the 20% of AFD excess mixture is mainly composed of chain segments with partially reacted hardener units. In the first case, the crosslinking density is higher due to the increased chemical conversion. However, the partially reacted units of the 20% of AFD excess lead to a higher molecular weight between crosslinks, which reduces the crosslinking density, as already demonstrated in previous studies [31,35]. As a result, the tensile strength and modulus decrease while the ductility is improved [43,44] in the last case.

On the other hand, the 0.2 wt.% CNT content shows a similar behavior, although there is less variation among the different AFD contents. This can be explained by a more CNT-governed network that may hinder the AFD influence due to its stiffening effect and the presence of possible CNT aggregates that rule the tensile strength.

It should be noted that the mechanical properties obtained are similar or higher than those of vitrimers and thermosets described in the recent literature. Li et al. [45] obtained a vitrimeric thermoset from DGEBA, polypropylene glycol diglycidylether (PPGDGE), and 2-AFD, which exhibited a tensile strength of 33.4 MPa. Similarly, Esmaeili et al. [46] achieved a tensile strength of 53 MPa by using an epoxy resin composed of DGEBA and XB3473 hardener with a 0.5 wt.% CNT. This demonstrates the superior mechanical performance capabilities of the CNT-doped vitrimers in comparison to conventional vitrimers and CNT-doped thermosets.

**Figure 5:**
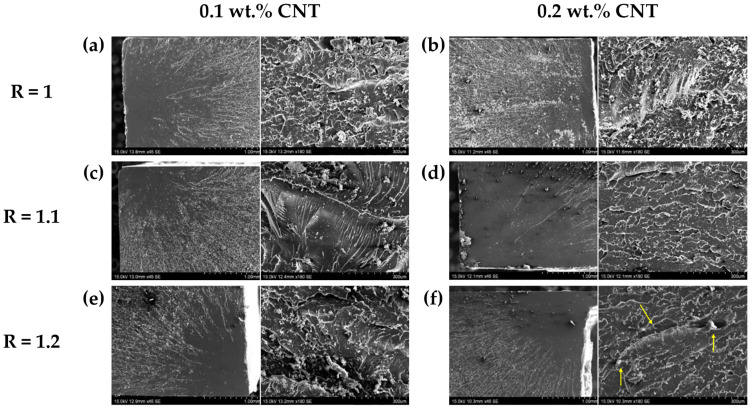
SEM images of (**a**) R = 1, (**c**) R = 1.1, and (**e**) R = 1.2 AFD ratios with a 0.1 wt.% CNT content, and (**b**) R = 1, (**d**) R = 1.1, and (**f**) R = 1.2 (microcracks arrowed) AFD ratios with a 0.2 wt.% CNT content.

The SEM images displayed in Figure 5 illustrate the morphology of the fracture surface after tensile testing. For the 0.1 wt.% CNT content, at R = 1, the fracture progressed rapidly and presented a bigger plain fracture surface (Figure 5a). At R = 1.1, a river pattern is observed on the fracture surface with interleaved plain surface regions (Figure 5c). Finally, at R = 1.2, a hackle pattern appears (Figure 5e). The two latest patterns indicate a slower crack propagation in which the energy consumption increases as the fracture progresses, which is in good agreement with the previous statements since the failure strain increased with the AFD content for this CNT condition. For the 0.2 wt.% CNT content, there is a similar fracture progression behavior, without any apparent changes between the AFD contents (Figure 5b,d,f), and a rapid fracture with a plain surface, corroborating the mechanical properties obtained in Table 2.

**Figure 6:**
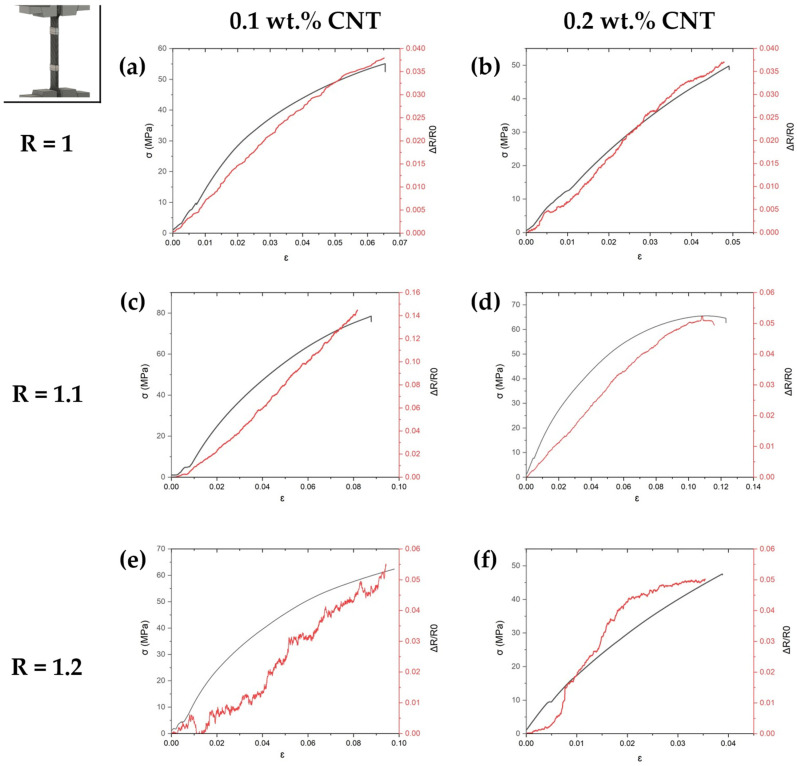
Load–strain and resistance–strain curves of tensile tests of (**a**) R = 1, (**c**) R = 1.1, and (**e**) R = 1.2 AFD ratios with a 0.1 wt.% CNT content, and (**b**) R = 1, (**d**) R = 1.1, and (**f**) R = 1.2 AFD ratios with a 0.2 wt.% CNT content.

Regarding the strain-sensing capabilities, the pattern of the electromechanical curves is very similar at low AFD contents, showing, in these cases plotted in Figure 6a–d, linear behavior. This indicates a prevalence of in-contact mechanisms over tunneling effect ones, which would be reflected in a linear–exponential variation in the electrical resistance with applied strain [47].

**Figure 7:**
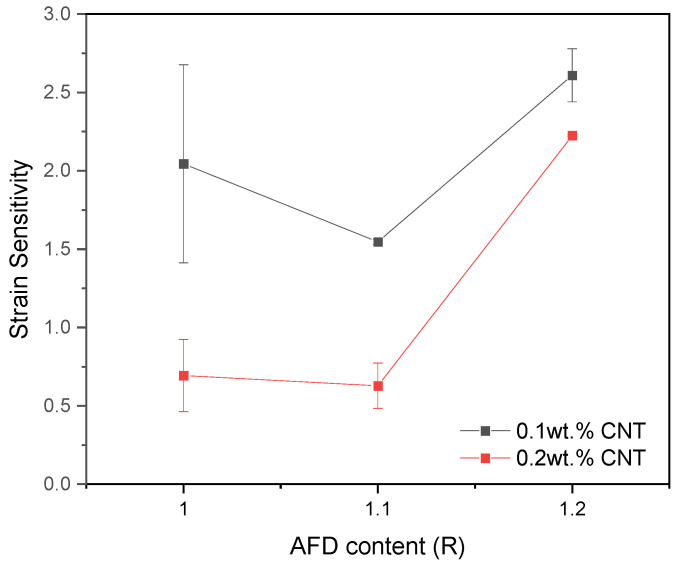
Strain sensitivity as a function of the AFD content for the tensile tests.

The GF is summarized in Table 2 and Figure 7 as an average of the deformation values at the linear region for all the experimental systems except for the 20% AFD excess system with 0.2 wt.% CNT, which corresponds to the average of the deformation values between 1 and 2%. In this regard, a detriment of GF is observed when increasing the CNT content. As the CNT content increases to 0.2 wt.%, the electrical conductivity increases due to the formation of a higher number of electrical pathways, and the electrical behavior becomes mainly governed by the contact between adjacent CNTs, which is almost invariable with applied strain, so that the GF decreases [47]. The effect of AFD content is significantly more complex and depends on the CNT concentration. At 0.1 wt.%, there is a decrease in the GF that may be explained by a more interconnected CNT network, with a higher presence of contact mechanisms, which was reflected by a higher electrical conductivity.

However, increasing the AFD content to 20% of excess leads to a higher gauge factor and results in a linear–exponential variation in the electrical behavior with applied strain, as shown in Figure 6e,f. This may be due to the presence of unreacted hardener domains, as previously explained, which may cause material segregation into regions with CNT clusters and regions with lower nanoreinforcement content [48,49], although both are well dispersed without significant CNT agglomerations. The overall electromechanical behavior is governed by regions with a low CNT concentration, where tunneling mechanisms predominate over contact and intrinsic mechanisms of the regions with a higher CNT concentration [50]. These tunneling mechanisms present a higher sensitivity to mechanical deformation, which would explain the increasing GF.

The results obtained in this study showed similar GF values to strain sensors based on CNT-doped epoxy polymers, which are typically in the range between 0.3 and 2.9 [46,51,52]. This demonstrates that the introduction of dynamic covalent networks with the AFD hardener does not affect the sensitivity of epoxy polymers.

#### 3.2.2. Bending Tests

**Figure 8:**
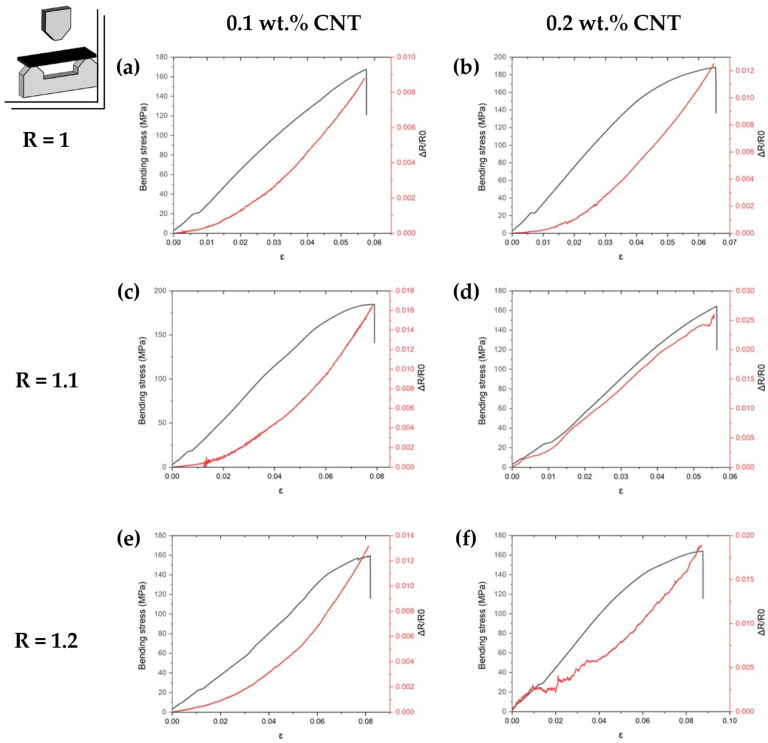
Load–strain and resistance–strain curves of three-point bending tests of (**a**) R = 1, (**c**) R = 1.1, and (**e**) R = 1.2 AFD ratios with a 0.1 wt.% CNT content, and (**b**) R = 1, (**d**) R = 1.1 and (**f**) R = 1.2 AFD ratios with a 0.2 wt.% CNT content.

Table 3 and Figure 8 summarize the electromechanical response under bending load. Here, the mechanical behavior matches the behavior of the tensile tests, with a drop in the mechanical properties with a 20% AFD excess due to lower crosslinking density, which promotes a lower modulus, flexural strength, and a higher ductility of the polymer for the 0.1 wt.%; and with the predominance of the CNT stiffening effect over the AFD influence is more prevalent with a higher CNT content. Thus, at higher CNT contents, the AFD content does not produce a significant variation of the mechanical properties. In this regard, a slight increase in flexural strength is observed, mainly attributed to a crack-bridging effect of the CNTs in combination with their highly stiffening effect. Moreover, as previously seen in the tensile tests, the results demonstrate superior mechanical capabilities in terms of flexural strength when compared to conventional epoxy resins. Here, Meng et al. [53] developed an epoxy resin composed of DGEBA and diamino diphenyl-sulfone (DDS) as a reference thermoset and achieved a flexural strength of 100.3 MPa, which is significantly lower than the values obtained in this study.

**Figure 9:**
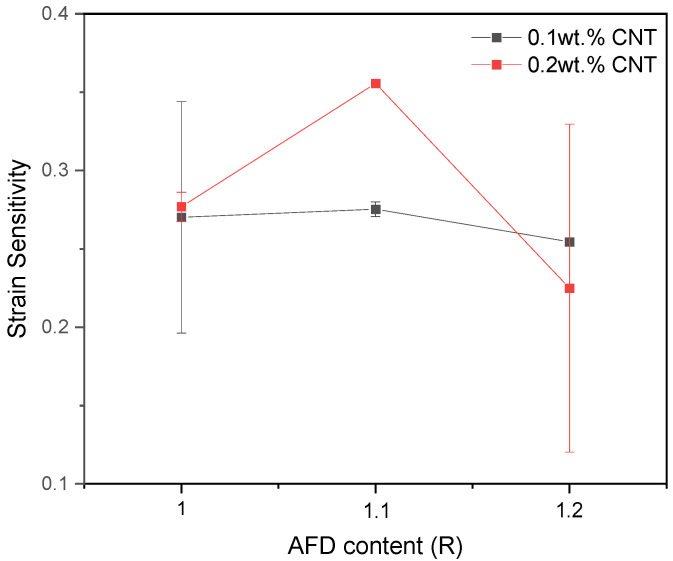
Strain sensitivity as a function of the AFD content for the three-point bending tests.

Regarding the strain sensitivity, a linear behavior of the electrical resistance with applied strain is observed (Figure 8), due to the previously mentioned prevalence of contact mechanisms over tunneling ones. Furthermore, a decrease in the GF is observed at bending conditions in comparison to tensile ones, as shown in the average GF for the deformation values at the linear regions in Table 3 and Figure 9. This may be explained by the compressive effect of the upper surface during the bending tests, which promote a reduction in the overall sensitivity of the sample in comparison to a pure tensile test [47]. However, the obtained values are within the abovementioned sensitivity range of CNT-doped epoxy sensors [46,51,52].

## 4. Conclusions

The main objective of this study was to investigate the correlation between the electromechanical behavior of a self-healing epoxy resin and the structure provided by different ratios of the dynamic curing agent based on disulfide bonds, together with the concentration of carbon nanotubes. The resulting polymeric networks were analyzed in terms of their electrical, mechanical, and electromechanical properties.

The electrical conductivity analysis showed that the higher the CNT concentration, the higher the electrical conductivity due to a higher number of electrical pathways. However, the AFD content provided two opposite effects: on the one hand, at low CNT contents, the presence of unreacted AFD domains actuating as insulating areas decreased the electrical conductivity; on the other hand, at high CNT contents, the higher viscosity of the AFD mixture induced a lower sedimentation during curing, providing a higher electrical conductivity.

In terms of mechanical properties, the tensile and three-point bending tests provided similar results. The mechanical behavior of the R = 1.1 AFD system with a 0.1 wt.% CNT showed a 45% increase in tensile strength when compared to the stoichiometric mixture due to improved chemical conversion. However, a higher AFD content (R = 1.2) led to a 15% reduction in tensile modulus and a 41% reduction in tensile strength, together with a 36% higher ductility. These phenomena were associated with an increased number of unreacted hardener units that lowered the crosslinking density. The 0.2 wt.% CNT systems did not show differences regarding the mechanical properties between the different AFD contents due to the toughening effect of the carbon nanotubes, thus increasing the tensile modulus and strength when compared to the 0.1 wt.% CNT systems.

Regarding the electromechanical properties, the stoichiometric and R = 1.1 AFD systems provided a linear behavior when correlating electrical resistivity with applied strain. The sensitivity was found to be inversely proportional to the electrical conductivity. In this regard, a higher CNT content leads to a decreased sensitivity since the electrical behavior becomes mainly governed by the contact between adjacent CNTs. However, increasing the AFD content to 20% excess with a 0.2 wt.% CNT leads to a higher gauge factor with a linear–exponential variation in the electromechanical behavior due to the predominating tunneling mechanisms of the regions with a lower CNT concentration.

Finally, it must be noted that all the experimental systems provided an excellent electrical response with the applied strain, mimicking the stress–strain curve. This demonstrates the potential use of self-healing vitrimers as the matrices of composite materials with an integrated SHM structure. Functional structures composed of these materials, such as offshore wind generators or aerospace structures, would be capable of detecting damages throughout their volume and autonomously repairing any discontinuities to recover their original properties. In the case of suffering any damage, a programmed system will detect an increase in electrical resistance and trigger an autonomous increase in voltage. This will increase the temperature via the Joule effect and repair the damage. Therefore, further investigation is needed to explore the interaction between reinforcement and smart vitrimers in terms of their electromechanical behavior and self-healing capabilities, which will contribute to the development of smart composites for future industrial implementation.

## Figures and Tables

**Table 1:** Parameters of three-roll-milling process.

Cycle	Gap 1 (μm)	Gap 2 (μm)	Rotating Speed of the Last Roll (rpm)
1	120	40	250
2	75	25	250
3	45	15	250
4–7	15	5	250

**Table 2:** Mechanical and strain-sensitivity values of the tensile test.

CNT Content	Epoxy/AFD Ratio	Tensile Modulus (GPa)	Tensile Strength (MPa)	Failure Strain (%)	Gauge Factor
0.1 wt.%	R = 1	1.68 ± 0.13	55.15 ± 0.72	7.54 ± 0.89	2.05 ± 0.63
	R = 1.1	1.7 ± 0.21	80.18 ± 3.19	8.6 ± 2.14	1.55 ± 0.02
	R = 1.2	1.44 ± 0.46	47.37 ± 13.41	11.71 ± 3	2.61 ± 0.17
0.2 wt.%	R = 1	1.81 ± 0.09	60.46 ± 7.01	7.12 ± 1.88	0.69 ± 0.23
	R = 1.1	1.78 ± 0.36	73.3 ± 7.35	7.03 ± 1.08	0.63 ± 0.15
	R = 1.2	1.81 ± 0.18	51.44 ± 8.27	5.5 ± 2.45	2.22 ± 0.01 *

* This sensitivity corresponds to the average GF for deformation values between 1 and 2%.

**Table 3:** Mechanical and strain sensitivity values of the three-point bending test.

CNT Content	Epoxy/AFD Ratio	Flexural Modulus (GPa)	Flexural Strength (MPa)	Failure Strain (%)	Gauge Factor
0.1 wt.%	R = 1	2.51 ± 0.76	177.26 ± 14.32	7.09 ± 1.41	0.27 ± 0.07
	R = 1.1	2.36 ± 0.2	185.63 ± 18.26	8.16 ± 1.13	0.28 ± 0.01
	R = 1.2	1.50 ± 0.51	142.17 ± 16.87	8.61 ± 3.09	0.26
0.2 wt.%	R = 1	2.96 ± 0.57	191.97 ± 5.2	6.73 ± 0.36	0.26
	R = 1.1	2.45 ± 0.28	194.81 ± 28.91	7.97 ± 2.02	0.28
	R = 1.2	2.52 ± 0.43	183.91 ± 25.13	7.25 ± 1.85	0.24 ± 0.04

## Data Availability

The data presented in this study are available on request from the corresponding authors.
